# Comparative models of uveitis

**DOI:** 10.1038/s41433-025-03693-6

**Published:** 2025-02-18

**Authors:** Olivia S. Knutson, Soyoung Choi, Simon Williams, Virginia L. Calder

**Affiliations:** 1 Addenbrooke’s Hospital, Cambridge University Hospitals, Cambridge, UK; 2 UCL Institute of Ophthalmology, London, UK

**Keywords:** Inflammation, Uveal diseases

## Abstract

Several clinical subtypes of uveitis exist yet specific immunopathogenic mechanisms involved remain unclear. Ex vivo studies are limited by lack of fresh retinal biopsies and studies have relied on aqueous humour or peripheral blood, which may not directly reflect disease. The aim of this review is to compare the various in vivo models and review their contributions to our understanding of disease processes. These models, although unable to reflect all clinical signs, have provided insight into the contribution of genes and molecules, characterisation of effector T-cells, cell trafficking into retinal tissues, the contribution of tissue-resident myeloid cells and the mechanism(s) of action of several anti-inflammatory compounds. In vivo uveitis models have provided an excellent resource with which to study the molecular and cellular processes involved. Recent refinements in models, improved imaging, and the application of omics have greatly increased the number of readouts and translational opportunities. Future approaches with in vitro models will also be discussed.

## Introduction

Uveitis, including both infectious and non-infectious forms, describes a range of inflammatory conditions affecting the uveal tissues. In this review we have focussed on non-infectious uveitis (NIU) which affects people of working age, for whom currently available treatments are inadequate. NIU describes more than 30 distinct clinical phenotypes, which can affect either the anterior or posterior segment, or both (pan uveitis). Disease can occur alone or as part of a systemic disease. A significant proportion of patients with anterior uveitis have an associated ankylosing spondylitis and are HLA-B27-positive [1] whereas many patients with posterior uveitis also suffer from sarcoidosis or Behçet’s disease [2]. Due to the complexities of disease classification, management and treatment stratification, several international study groups were created to reach a consensus on clinical guidelines and to facilitate multicentre studies. Hence the agreed definition of NIU is that it can be bilateral, inflammation involves the uveal tract (iris, ciliary body, choroid), and adjacent areas (vitreous humour, retina, optic nerve) are also affected. The pathology of disease determines treatment options, with immunosuppressives such as corticosteroids and/or cyclosporin A often recommended for severe, sight-threatening disease [3]. However, NIU is extremely heterogenous in its clinical severity and duration, and it is difficult to predict who will respond to immunosuppressive treatment.

## Investigating immune pathways in NIU

Due to the associated risks, obtaining fresh biopsies from untreated NIU patients with active disease is not feasible. End-stage, fixed ocular specimens can be studied to confirm expression of specific molecules, but results might not reflect ongoing disease pathways. Furthermore, donors will have likely received many different forms of anti-inflammatory treatments over several years, making it difficult to relate specific findings to disease activity.

For patient studies, peripheral blood is often used to investigate immune cells. Ideally this approach should include clinically well-defined, treatment-naïve patients to provide baseline levels. Small volumes of intraocular fluid specimens – aqueous and vitreous – have also been collected, often during cataract surgery or vitrectomy, for use in protein- and cell-based studies. However, whilst such data can be informative, it represents only a single timepoint in the course of disease.

Experimental animal models have therefore been an invaluable tool for studying intraocular inflammation and immune mechanisms at different stages of disease. They enable the identification of intraocular and peripheral cell populations, and responses to treatment. Many animal species have been demonstrated to be inducible for experimental autoimmune uveitis (EAU), although uveitis does not occur spontaneously in the wild. Most preclinical studies have involved rats and mice, where there are similarities with man in respect of ocular anatomy, immune mechanisms and in the role of the blood-retinal barrier in controlling entry of cells and proteins from the periphery into the retina.

## In vivo models of anterior uveitis

Anterior uveitis can be a clinically severe, sight-threatening form of disease. Inflammation affects the anterior segment and disease can vary in duration. In vivo rodent models have usually involved immunisation with lipopolysaccharide (endotoxin), often given intravitreally, which results in an acute inflammation affecting the iris and ciliary body which lasts 24–48 h, termed endotoxin-induced uveitis (EIU). Within 16–24 h’ post immunisation a wave of granulocytes, predominantly neutrophils, infiltrate the ocular tissues in response to signalling pathway activation via the endotoxin receptor, TLR-4. This model offers an extremely narrow timeframe with which to investigate disease mechanisms or to administer therapies, and most studies involve pre-treating rodents with potential anti-inflammatory agents prior to inducing EIU [4, 5].

A model of acute anterior uveitis was developed in Fischer 344 rats immunised with bovine ocular melanin (EMIU). This was found to be CD4^+^T cell mediated as it was inhibited by anti-CD4 antibody. The disease entered remission at 4 weeks’ post induction and was associated with subsequent spontaneous relapses [6]. EMIU therefore holds greater potential as a preclinical model for anterior uveitis.

## Evidence for autoimmune pathways in posterior uveitis

Initially the evidence for posterior uveitis having an autoimmune aetiology was due to it sharing many features with more established autoimmune diseases, including multiple sclerosis, type 2 diabetes, and rheumatoid arthritis. The observed infiltration of CD4^+^T cells into retinal tissues, an upregulation of HLA-DR expression by tissue-resident cells, and the clinical efficacy of the T-cell inhibitor cyclosporin A all suggested posterior uveitis to be an autoimmune disease. Further evidence arose from the development of the in vivo model EAU in which purified retinal antigens emulsified in adjuvant were administered to naïve animals, resulting in development of a localised intraocular inflammation [7]. The specific autoantigen(s) involved in posterior uveitis remain unclear despite considerable research in this area, and therefore posterior uveitis is usually referred to as being an immune-mediated retinal inflammatory disease.

## In vivo models of posterior uveitis

Models of EAU induced by uveitogenic retinal proteins or peptides in mice and rats share similar immunological mechanisms and pathological characteristics, although the progression and severity of the disease varies, based on species-, strain- and dose-dependent differences [8]. Purified retinal antigens used for inducing EAU include S-Ag (arrestin), interphotoreceptor binding protein (IRBP), rhodopsin/opsin, phosducin, and recoverin. For inducing EAU in inbred mice IRBP is the antigen commonly used and, by comparing sequential IRBP peptide sequences, C57BL/6 mice mainly responded to peptide 1–20, whilst other mouse strains developed EAU in response to different sequences [9], highlighting that genes involved in antigen presentation to CD4^+^T cells (MHC Class 2) determine responsiveness to specific retinal antigens. The time course and severity of EAU development also varies; B10RIII mice develop an acute and severe form of EAU 7–10 days post immunisation and inflammation persists for 14–20 days, whereas C57BL/6 J mice exhibit a milder form of EAU, detectable by 14–17 days which peaks on day 20–23 and then declines, but with low levels of retinal inflammation still detectable on day 75. The C57BL/6 model can therefore be used for more translational studies as the inflammation is reversible and there is an extended timeframe with which to introduce any therapy post EAU induction.

Another method for inducing EAU involves adoptive transfer (AT), where retinal antigen-specific, activated CD4^+^T cells are administered to naïve mice. AT has several advantages as it avoids the need for adjuvants or pertussis in the immunisation protocol. However, it often takes longer to develop, is monophasic, and clinically less severe. When originally developed, AT provided the definitive evidence that CD4^+^T cells induce EAU, whereas CD8^+^T cells, B cells or serum could not [10]. More recently AT has been used to demonstrate the differential capacity of CD4^+^T cell subsets to induce EAU (described below).

There are also spontaneous models of EAU using transgenic mice. Several studies have involved the IRBP-specific T cell receptor-transgenic mouse (R161H-/-) in B10RIII mice [11]. Another spontaneous model uses the Autoimmune Regulator (AIRE)(-/-) genetically deficient mice [12]. These models are more reflective of human disease in terms of their spontaneousness, and each model exhibited a different clinical progression with persistent cellular infiltrates in the R161H model, suggesting a more translational model for human disease. A spontaneous relapsing/remitting model (R14) has also been described in rats immunised with an IRBP peptide in which oral tolerance of the same peptide was demonstrated to effectively downregulate the disease [13].

A chronic model of EAU has recently been described in which peptides 1–20 and 161–180 were co-administered, resulting in disease being detected at 16 weeks post immunisation. Furthermore, long-lived memory T cells (CD44^hi^, IL-7R^+^, IL-15R^+^, CD4^+^) were detected in retinal tissues 12–16 weeks post-EAU induction which, upon isolation, were still able to adoptively transfer disease to naïve mice [14]. These long-lived cells are thought to be responsible for the persistent low grade intraocular inflammation observed after disease resolution.

## Readouts of EAU

**Fig. 1:**
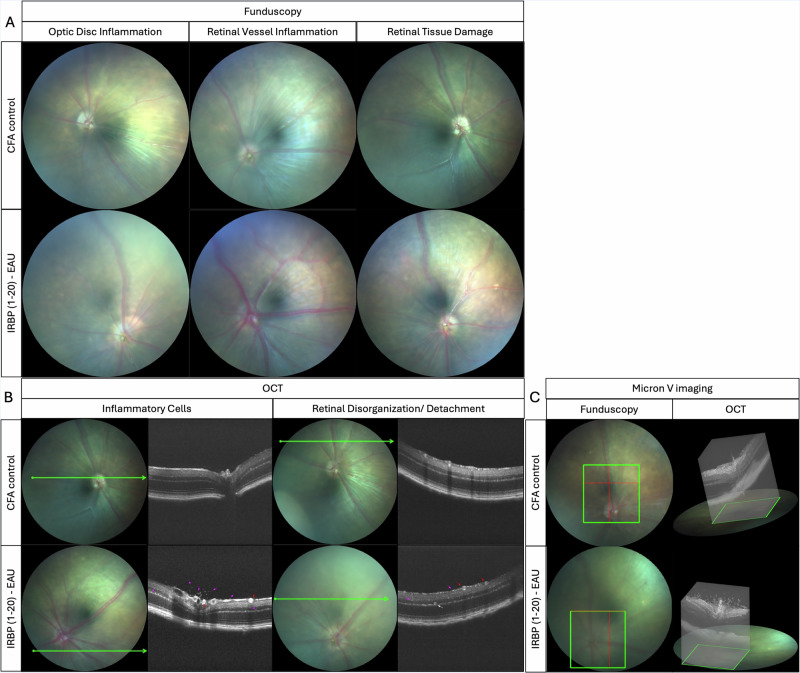
Live imaging of mouse retinal tissues in EAU and controls. Fundus (**A**), OCT (**B**) and 3D retinal slices (**C**) images using Micron V (Phoenix MICRON, Oregon, USA) to visualise characteristic retinal features in C57BL6/J female mice, comparing adjuvant (CFA) controls with IRBP1-20-induced EAU at days 14–21 post immunisation.

Rodent models of EAU have the crucial advantage of enabling a plethora of readouts that characterise the pathological features of the disease. Fundus photography can allow the visualisation of many EAU-induced features in C57BL6 mice. This includes optic disc inflammation, vasculitis and retinal tissue damage where significant changes can be seen from around 14–21 days post immunisation (DPI) in the IRBP 1–20 (Fig. 1) and IRBP 651-670 induced models [15–22]. Fluorescein angiography (FA) allows visualisation of leakage of fluorescein to mark features of increased vascular leakage, a feature of EAU [15, 19, 22, 23]. Optical coherence tomography (OCT) enables tracking of the changes in the organisation of retinal components and the thicknesses of retinal layers, where features including increased infiltration of inflammatory cells, as illustrated (Fig. 1), and retinal disorganisation and detachment can be observed in EAU mice [15, 18, 20, 21, 23, 24]. Electroretinography (ERG) identifies functional deterioration of the retina where scotopic (dark-adapted) and photopic (light-adapted) a- and b-wave amplitudes can be significantly reduced in the EAU mice [16, 18, 20, 25]. Despite the range of possible observations throughout the progression of EAU, investigations focus on in vivo readouts that answer research questions whilst considering the safety of animals with time spent under anaesthesia.

The possibilities of ex vivo observations in these models of EAU have also majorly expanded. Haematoxylin & eosin (H&E) retinal staining has been used to assess the severity of EAU; it can help to identify features like vasculitis, infiltrating immune cells (seen in parallel to OCT) and retinal layer disorganisation [15, 20, 21, 23, 25]. Immunostaining in the EAU retina has allowed more specific visualisation of a reduced amount of rod, cone, bipolar, amacrine, horizontal and retinal ganglion cells, and an increase of CD45^+^leucocytes and CD11b^+^myeloid cells [8, 15, 16, 23]. Immunohistochemical observations offer the advantage of visualising the organisation and morphology of cell types in different layers of the retinae through cross-sections or flat-mounted samples. However, these can often be more qualitative assessments with a limited number of markers at one time on a selected number of planes or layers.

Readouts assessed with flow cytometry (FC) offer a high-throughput analysis of quantitative data including cell size, granularity, and immunophenotyping with multiple antibodies on millions of cells. More recently, single-cell RNA sequencing (scRNA-seq) has provided high-resolution transcriptomic data, revealing cell heterogeneity and therefore rarer subsets in disease-associated populations.

## Pathogenic cells in EAU

Myeloid cells have an essential role in the retina, clearing debris/pathogens and in homoeostasis of retinal tissues. In conjunction with immunohistology, FC results from retinal cell populations have demonstrated increased levels of CD4^+^T cells, CD45^+^ leucocytes, CD11b^+^macrophages and MHC class 2^+^microglia [26–29].

A certain subset of these myeloid cells are macrophages which are tissue-resident (microglia) or derived from peripheral blood (monocyte-derived). Microglia are distinct in that they are an embryonic yolk sac-derived self-renewing population [30], predominantly inhabiting the inner retinal segment, migrating to the outer layer in response to inflammation and changing from ramified to amoeboid morphology in the process [31].

While monocytes share a common myeloid progenitor with microglia, monocytes are generated by bone marrow haematopoiesis during adulthood [32]. These monocytes can extravasate via the choroid into the outer retinal layer where they differentiate into macrophages, which show highly plastic morphology [33]. Monocyte-derived macrophages are typically classified into pro-inflammatory (M1) or homoeostatic (M2) roles, depending on their microenvironment and subsequent antibody and cytokine expression [34].

The precise role of yolk-sac derived microglia during retinal inflammation was investigated in EIU using single-eye mRNA-Seq and Cx3cr1 Cre mice to track microglia transcriptional changes over 2 weeks. It was thus demonstrated that these microglia resumed their homeostatic state 2 weeks after acute retinal inflammation [35]. Evidence for the role of retinal microglia in EAU has come from studies using scRNA-seq. Whilst it has been known that CC chemokines are heavily involved in the EAU pathogenesis, recent investigations have shown that blockage of CCR5/CCL5 interaction can have therapeutic potential by reducing infiltrating T-cells and microglial activation [8, 16].

The AT model of EAU had provided evidence that CD4^+^T cells initiate the EAU disease process and further studies have compared populations of retinal antigen-specific CD4^+^T cells [36]. Whilst both Th1 and Th17 cell subsets are uveitogenic, the course of disease varies, suggesting that each cell subset utilises different immune pathways. Further studies have also identified a significant increase of a unique subset of T cells - Th17/Th1 - in the retina, which expressed both IFNγ and IL-17, were significantly upregulated at peak disease and were resistant to the inhibitory effects of dexamethasone [37].

Analysis of retinal cell infiltrates identified multiple subsets of regulatory T cells (Treg) which modulate disease [38]. In a recent review, several EAU models, induced with different peptides and in different strains, have recently been compared to help select the most appropriate model to use for each research study [39].

## Applying EAU to understand role of human Treg

Whilst many studies of EAU have provided insights into human disease, some findings have been initially demonstrated in uveitis patients which have subsequently been confirmed in EAU. One example of this was the finding of increased levels of a subset of CD4^+^CD25^+^ FoxP3^+^ Tbet^+^ TIGIT^+^ Treg in peripheral bloods from NIU patients in clinical remission in comparison with active disease [40]. This Treg subset was subsequently investigated in a mouse model of EAU and it was reported that TIGIT stimulation, administered from day 20 onwards (active EAU) significantly suppressed disease scores and inhibited Th17 cell infiltration [41]. These findings suggested that certain subsets of Treg cells could be regulating the disease and might offer a potential therapy in man. In a previous clinical trial (UVEREEG; NCT024944929) [42], intravitreal administration of polyclonal Treg was performed in patients with bilateral severe forms of NIU. Whilst results were inconclusive, further understanding of the different Treg subsets could offer a future alternative immunosuppression for these patients.

## Organoids as models of uveitis

Organoids are multicellular, three-dimensional tissue cultures that recapitulate many aspects of the complex structure and function of the corresponding in vivo tissue [43]. These tissues are cellularly heterogeneous, in contrast to feeder cell based cultures, yet are more simplified than animal models [44].

Organoids therefore enable the investigation of the interaction of immune cells with epithelial cells in a reductionist manner [44]. They may be co-cultured with T cells to evoke immune-mediated epithelial cell differentiation [45, 46]. For example, the triple co-culture of epithelial cells, cytotoxic T cells and bacteria identified the H. pylori-induced expression of the checkpoint inhibitor programmed cell death ligand-1 [47].

In terms of ocular diseases, labs have grown optic cups and retinal organoids with emergent electrophysiological properties [48–50], as well as corneal organoids [51]. This technology has been successfully employed to model genetic diseases including Leber congenital amaurosis [52] and retinitis pigmentosa [53]. In the future, it is possible that these technologies could be applied to further elucidate the pathogenesis of uveitis.

## Conclusion

The in vivo models of uveitis and imaging methods have been extensively refined over the last 30 years, allowing investigators to clinically score disease temporally and identify several relevant effector cells and molecules. These models have therefore become extremely informative in preclinical studies. However, given their heterogeneity, it is crucial to select the model which best fits the research question. In addition, while many potential therapies have been demonstrated to be effective for reversing EAU, the results do not always translate to patients. Hence, effective and safe treatments for the various clinical subtypes of uveitis remain elusive.
